# Duration Mismatch Negativity Predicts Remission in First-Episode Schizophrenia Patients

**DOI:** 10.3389/fpsyt.2021.777378

**Published:** 2021-11-25

**Authors:** Suguru Nakajima, Yuko Higuchi, Takahiro Tateno, Daiki Sasabayashi, Yuko Mizukami, Shimako Nishiyama, Tsutomu Takahashi, Michio Suzuki

**Affiliations:** ^1^Department of Neuropsychiatry, Graduate School of Medicine and Pharmaceutical Sciences, University of Toyama, Toyama, Japan; ^2^Research Center for Idling Brain Science, University of Toyama, Toyama, Japan; ^3^Health Administration Center, Faculty of Education and Research Promotion, Academic Assembly, University of Toyama, Toyama, Japan

**Keywords:** remission, predicting, event-related potential (ERP), mismatch negativity (MMN), first-episode schizophrenia (FES)

## Abstract

**Objective:** Remission in schizophrenia patients is associated with neurocognitive, social, and role functioning during both the early and chronic stages of schizophrenia. It is well-established that the amplitudes of duration mismatch negativity (dMMN) and frequency MMN (fMMN) are reduced in schizophrenia patients. However, the potential link between MMN and remission has not been established. In this study, we investigated the relationship between MMNs and remission in first-episode schizophrenia (FES) and their association with neurocognitive and social functioning.

**Method:** dMMN and fMMN were measured in 30 patients with FES and 22 healthy controls at baseline and after a mean of 3 years. Clinical symptoms and cognitive and social functioning in the patients were assessed at the time of MMN measurements by using the Positive and Negative Syndrome Scale (PANSS), modified Global Assessment of Functioning (mGAF), Schizophrenia Cognition Rating Scale (SCoRS), and the Brief Assessment of Cognition in Schizophrenia (BACS). Remission of the patients was defined using the criteria by the Remission in Schizophrenia Working Group; of the 30 patients with FES, 14 achieved remission and 16 did not.

**Results:** Baseline dMMN amplitude was reduced in FES compared to healthy controls. Further, baseline dMMN in the non-remitters had decreased amplitude and prolonged latency compared to the remitters. MMN did not change during follow-up period regardless of parameters, diagnosis, or remission status. Baseline dMMN amplitude in FES was correlated with future SCoRS and PANSS total scores. Logistic regression analysis revealed that dMMN amplitude at baseline was a significant predictor of remission.

**Conclusions:** Our findings suggest that dMMN amplitude may be a useful biomarker for predicting symptomatic remission and improvement of cognitive and social functions in FES.

## Introduction

Symptom remission is thought to represent the principal target for psychopharmacological interventions in schizophrenia (1, 2), while the concept of clinical remission also consists of improvements in cognitive and social functioning during the course of the illness (3). There is a consensus that early intervention can lead to a higher rate of symptomatic remission and better functional outcomes in patients with schizophrenia, potentially by preventing and/or ameliorating active brain changes at the early stages of the illness (4, 5). However, current evidence supports that clinical and neurobiological factors associated with early neurodevelopmental pathology e.g., premorbid intelligence (6), obstetric complications (7), and gross brain morphology (8, 9) as well as genetic factors (10) may also contribute to worse functional outcomes and symptom severity in later stages of schizophrenia. In recent years, as described below, event-related potential (ERP) abnormalities are considered to be suitable biomarkers of functional recovery for schizophrenia (11) and clinical high risk (CHR) patients (12, 13). To our knowledge, however, very few studies to date have attempted a detailed examination of a potential link between ERP at early stages after onset and clinical remission (i.e., symptom remission and improvements in cognitive and social functions) in schizophrenia. Thus, further studies will be required to detect reliable biomarkers for predicting clinical and functional outcomes of schizophrenia, which may support treatment decisions based on the individual neurobiological differences.

Mismatch negativity (MMN) is ERP generated when a sequence of unattended repetitive standard stimuli is interrupted by a deviant stimulus (e.g., duration, frequency, and intensity) (14, 15). It is considered that MMN is generated by a fronto-temporal network associated with pre-attentive sensory processing (16). In schizophrenia patients, MMN impairment has been repeatedly reported and may reflect their N-methyl-D-aspartate (NMDA) receptor hypofunction (17, 18) at this network. The duration MMN (dMMN) deficit may occur in different psychotic disorders irrespective of their specific etiology and symptomatology (14). However, previous studies have suggested the role of MMN as a “breakthrough biomarker” for schizophrenia (19); it has reduced amplitude with larger effect size than other psychiatric disorders such as bipolar disorder (20), remains stable over time, and is independent of state-related changes (21). In particular, reduced amplitude of dMMN exists in various stages of psychosis, including CHR status before onset and both first-episode and chronic stages of schizophrenia (22–24), as a rather stable vulnerability marker and also reflects cognitive and social functions in various clinical conditions (25, 26). It has been demonstrated that high baseline amplitude of dMMN in CHR individuals is associated with functional and symptomatic improvement regardless of psychosis onset (27, 28), potentially implicating its role as a predictor of remission in patients with psychotic disorders. A recent literature has also reported that a decrease in baseline MMN amplitude in first episode psychosis was a significant predictor of subsequent treatment resistance (28). Regarding remission, Kim et al. (11) demonstrated that baseline amplitude of dMMN at the frontal site predicted short-term (i.e., after 6 months from baseline) symptomatic remission in chronically medicated patients with schizophrenia. However, this finding needs replication in patients with fewer confounding factors (especially illness chronicity and medication) and longer clinical follow-up to clarify the potential utility of baseline MMNs as biomarkers to predict prognosis. Furthermore, it remains unknown whether an active decline in MMN amplitude demonstrated during the early course of schizophrenia (23, 29, 30) could be associated with a later clinical course.

In this study, we investigated the relationship between MMNs and symptom remission in first-episode schizophrenia (FES) and their relationship with neurocognitive and social functions. Based on the literature, we hypothesized that preserved baseline dMMN amplitude would be associated with symptomatic remission and better cognitive and social functions at follow-up in patients with FES. We also examined the relationship between frequency MMN (fMMN), which may be a less sensitive marker of schizophrenia than dMMN (12) but likely reflects cognitive functioning at later stages in CHR individuals (22), and remission in FES patients given that this has yet to be reported. Moreover, we explored potential differences in longitudinal MMN changes between the FES patients with and without symptomatic remission.

## Materials and Methods

### Participants

A total of 30 patients with schizophrenia (14 male and 16 female patients; mean age ± standard deviation, 23.5 ± 8.7 years old), recruited from the University of Toyama Hospital, participated in this study. Patients with schizophrenia were diagnosed by experienced psychiatrists based on the ICD-10 research criteria (31). Based on previous literature, only patients with FES with an illness duration of <2 years and a single psychotic episode were enrolled (32, 33). All patients received MMN measurements and clinical assessments, as described below, at least twice [once at baseline (Time 1) and again at follow-up (Time 2)], with a mean interval of approximately 3 years. In the meantime, between Time 1 and Time 2, all patients regularly received routine clinical observation once or twice a month by us or local psychiatric hospitals/clinics. Information on psychiatric and treatment history was collected from interviews with participants and their families or medical records. Eligible patients were confirmed to have a good hearing ability and good physical health based on physical examination and standard laboratory tests. Exclusion criteria for patients were: a history of substance abuse or dependence, seizures, head injury, and an estimated premorbid IQ of <70 based on the Japanese Adult Reading Test (34). Of the 30 patients with FES, 23 received antipsychotic medication (3.32 ± 3.34 mg/day, risperidone equivalent). A total of 22 healthy controls (H) (14 male and 8 female participants; mean age, 23.4 ± 4.2 years) were recruited from the community, university students, and hospital staff. Participants were screened for past or current Axis I disorders based on the Structured Clinical Interview for DSM-IV (SCID) (35). Additional exclusion criteria (in addition to those listed above) were: a history of psychiatric disorders in participants or their first-degree relatives. Demographic data at baseline evaluation are presented in Tables 1, 2.

**Table 1:** Demographic and clinical data for groups H and FES.

	***H***	**FES**	**Group difference^a^**
	***n* = 22**	***n* = 30**	
Age [years]	23.43 (4.21)	23.48 (8.67)	*P* = 0.98
Gender (male/female)	14/8	14/16	χ2= 1.47, *P* = 0.23
Follow-up period [years]	2.05 (1.25)	2.95 (2.29)	*P* = 0.10
JART	109.86 (5.93)	97.30 (10.56)	*P* < 0.01^**^

*All values are shown as means (standard deviations). H, healthy controls; FES, first episode schizophrenia; JART, Japanese Adult Reading Test*.

a *Demographic differences between groups were examined by chi-square or t-test*

(^**^P < 0.01).

**Table 2:** Demographic and clinical data for groups R and NR.

	**R**	**NR**	**Group difference^a^**
	***n* = 14**	***n* = 16**	
Age [years]	23.56 (6.31)	23.40 (10.07)	*P* = 0.96
Gender (male/female)	5/9	9/7	χ2= 1.27, *P* = 0.26
Follow-up period [years]	3.10 (2.47)	2.81 (2.03)	*P* = 0.75
JART	96.07 (9.59)	98.38 (10.93)	*P* = 0.56
Age at onset [years]	23.56 (6.31)	23.39 (10.07)	*P* = 0.95
Duration of illness [years]	0.57 (0.41)	0.58 (0.49)	*P* = 0.93
Duration of untreated psychosis [years]	0.16 (0.97)	0.03 (0.84)	*P* = 0.72
Duration of medication at baseline [years]	0.42 (1.07)	0.62 (1.08)	*P* = 0.63
**Antipsychotic dose (mg/day, risperidone equivalent)**			
Baseline (Time 1)	2.71 (2.55)	3.84 (3.73)	*P* = 0.36
Follow-up (Time 2)	2.73(1.64)	4.91(3.43)	*P* = 0.045^*^
**RSWGcr**			
Baseline (Time 1)	23.21 (5.13)	23.27 (5.21)	*P* = 0.98
Follow-up (Time 2)	10.71 (2.40)	18.63 (3.10)	*P* < 0.01^**^
**PANSS**			
**Baseline (Time 1)**			
:Positive symptoms	17.89 (5.13)	14.40 (5.77)	*P* = 0.12
:Negative symptoms ^b^	17.00 (6.07)	20.53 (5.58)	*P* = 0.13
:Global psychopathology	34.86 (7.73)	34.33 (8.47)	*P* = 0.87
:Total	69.64 (16.18)	69.27 (14.17)	*P* = 0.95
**Follow-up (Time 2)**			
:Positive symptoms	8.07 (1.62)	11.43 (3.64)	*P* < 0.01^**^
:Negative symptoms ^b^	10.50 (2.82)	17.25 (3.72)	*P* < 0.01^**^
:Global psychopathology	20.07 (2.25)	27.38 (5.97)	*P* < 0.01^**^
:Total	38.64 (4.82)	56.06 (8.20)	*P* < 0.01^**^
**Change**			
:Δ positive symptoms	−9.71 (5.55)	−3.00 (5.05)	*P* < 0.01^**^
:Δ negative symptoms	−6.50 (5.86)	−3.53 (5.69)	*P* = 0.19
:Δ global psychopathology	−14.79 (8.31)	−6.60 (9.53)	*P* = 0.025^*^
:Δ total	−31.00 (17.94)	−13.13 (17.30)	*P* = 0.014^*^
**BACS-Composite score**			
Baseline (Time 1)	−1.23 (0.81)	−1.50 (0.90)	*P* = 0.43
Follow-up (Time 2)	−0.97 (0.63)	−1.00 (0.84)	*P* = 0.93
ΔBACS-Composite score	0.26 (0.77)	0.50 (0.62)	*P* = 0.38
**SCoRS^c^**			
Baseline (Time 1)	6.14 (2.64)	5.40 (1.97)	*P* = 0.40
Follow-up (Time 2)	3.21 (1.93)	5.57 (1.87)	*P* < 0.01^**^
ΔSCoRS	−2.93 (2.55)	0.16 (2.35)	*P* < 0.01^**^
**mGAF^d^**			
Baseline (Time 1)	32.64 (6.64)	37.42 (8.66)	*P* = 0.14
Follow-up (Time 2)	51.43 (8.24)	38.88 (8.64)	*P* < 0.01^**^
ΔmGAF	18.79 (10.79)	0.66 (8.47)	*P* < 0.01^**^

*All values are shown as means (standard deviations). R, schizophrenia remitter; NR, schizophrenia non-remitter; JART, Japanese Adult Reading Test; RSWGcr, Remission in Schizophrenia Working Group criteria; PANSS, Positive and Negative Syndrome Scale; BACS, Brief Assessment of Cognition in Schizophrenia; SCoRS, Schizophrenia Cognition Rating Scale; mGAF, modified Global Assessment of Functioning*.

a *Demographic differences between groups were examined by chi-square or t-test (*

* *P < 0.05*,

** *P < 0.01)*.

b *A repeated measures ANOVA with group and time as between-subject variables showed no group-by-time interaction [F_(1, 25)_ =1.78, P = 0.19], demonstrating that improvement in negative symptoms did not differ between the NR and R groups*.

c *Data are ranging from 0 to 10, with larger number representing more worse function*.

d *Healthy subjects generally have a score ranging from 90 to 100*.

The study was conducted in accordance with the Declaration of Helsinki. The Committee on Medical Ethics of Toyama University approved the present study (No. I2013006) on February 5, 2014. After providing a full explanation of the purpose and procedures of the study, written informed consent was obtained individually from each study participant. For participants under 20 years of age, written consent was also received from a parent or guardian.

### Clinical Assessment

Clinical symptoms, cognitive function, and social function in patients with FES were evaluated by experienced psychiatrists or psychologists using the Positive and Negative Syndrome Scale (PANSS) (36), Brief Assessment of Cognition in Schizophrenia (BACS) (37, 38), Schizophrenia Cognition Rating Scale (SCoRS) (39–41), and modified Global Assessment of Functioning (mGAF) (42). BACS composite score was calculated by averaging the z-scores of the six primary BACS measurements (38). Clinical assessments were performed on the same day or within 2 weeks of EEG recordings.

### Assessment of Remission

The criteria by the Remission in Schizophrenia Working Group (RSWGcr) (43) were used to assess symptomatic remission in patients with FES. The RSWGcr score was defined based on ratings of eight focal symptoms on positive, negative, and general psychopathology subscales of the PANSS (P1, P2, P3, N1, N4, N6, G5, and G9) to determine the clinical remission of patients with schizophrenia (43). For symptomatic remission, maintenance over a 6-month period of simultaneous ratings of mild or less ( ≤ 3 points) on all items was required. Based on RSWGcr scores at Time 2 and those measured more than 6 months before Time 2, patients were defined as “remitter (R)” if both scores fulfilled symptomatic remission criteria and “non-remitter (NR)” if both did not. Patients with FES who did not meet the aforementioned definition were excluded from the current study. In this study, 14 patients met the remission criteria (R), whereas 16 did not (NR).

### MMN Recordings

MMNs were recorded using an auditory oddball paradigm based on an established method (32, 33, 44). EEG recordings were obtained with a Nihon Kohden EEG device (EEG-1250 version 07-02, Nihon Kohden Corp.) and a 32-channel Electrocap (Electrocap Inc.) or a 32-channel MCS cap (Medical Computer Systems Ltd.) in a wave-shielded and sound-attenuated room. Auditory stimuli were delivered binaurally through headphones with interstimulus intervals of 500 ms while participants were seated watching a silent cartoon. Two auditory oddball paradigms using duration and frequency deviant stimuli were employed. For dMMN, 1,500 stimuli consisting of 90% standard tones (1,000 Hz, 50 ms) and 10% deviant tones (1,000 Hz, 100 ms) were used. For fMMN, 1,500 stimuli consisting of 90% standard tones (1,000 Hz, 50 ms) and 10% deviant tones (1,500 Hz, 50 ms) were used. The auditory parameters were delivered at a 60-dB sound pressure level, 10 ms rise/fall time. Data were collected with a sampling rate of 500 Hz. The bandwidth was set at 0.53–120 Hz with a 60 Hz notch filter. The reference electrode was Aav and the ground electrode was *Z*. Electrode impedance was <10 kΩ. Averaging of MMN waves was performed using EPLYZER II software (Kissei Comtec, Co. Ltd.). Epochs were 600 ms for dMMN and 500 ms for fMMN, including a 100 ms pre-stimulus baseline. Artifacts (e.g., blinks, eye movements, and body movement) were manually rejected before the study participants were grouped. Next, EEG responses with deviant tones and standard tones were averaged off-line. After this process, 243.0 ± 81.8 (mean ± standard deviation) standard tones and 81.6 ± 24.4 deviant tones remained; the number of available epochs was lower in the FES group compared to the H group (*P* = 0.01, student's *t*-test) but did not differ between the R and NR groups (*P* = 0.18). Finally, an MMN waveform was obtained by subtracting standard waveforms from deviant waveforms. All pre-stimulus amplitudes were averaged at 50 data sampling points (from −100 to 0 ms, sampling rate: 500 Hz) and were defined as the average as zero-point. The amplitude and latency of dMMN and fMMN were used as parameters. For dMMN, the peak observed 130–250 ms after the start of the sound, and for fMMN, the peak observed 60–180 ms after the start of the sound was used as its amplitude (zero-point to peak) and latency (0 ms to peak). For the statistical analyses, only the recording at Fz, which generally has the greatest amplitude compared with other electrodes, was used as a representative of MMN for each individual, as reported previously (16, 17, 45).

### Statistical Analysis

**Figure 1:**
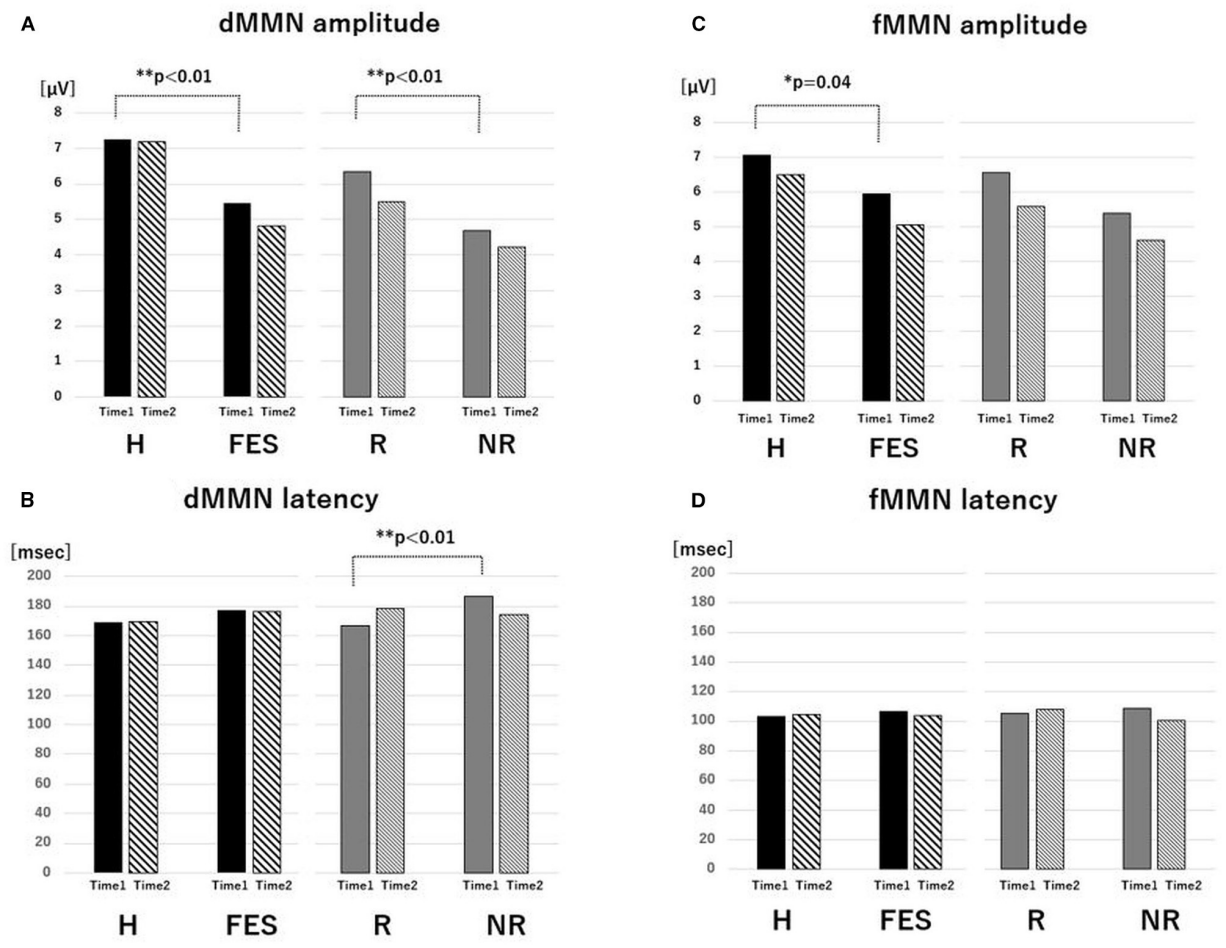
Value of MMN parameters. The average of amplitude **(A)** and latency **(B)** of dMMN and the average of amplitude **(C)** and latency **(D)** of fMMN for the H, FES, R, and NR groups for Time 1 and Time 2. *P < 0.05, **P < 0.01, ANCOVA. Abbreviations: dMMN, duration mismatch negativity; fMMN, frequency mismatch negativity; H, healthy control; FES, first episode schizophrenia; R, schizophrenia remitter; NR, schizophrenia non-remitter.

**Figure 2:**
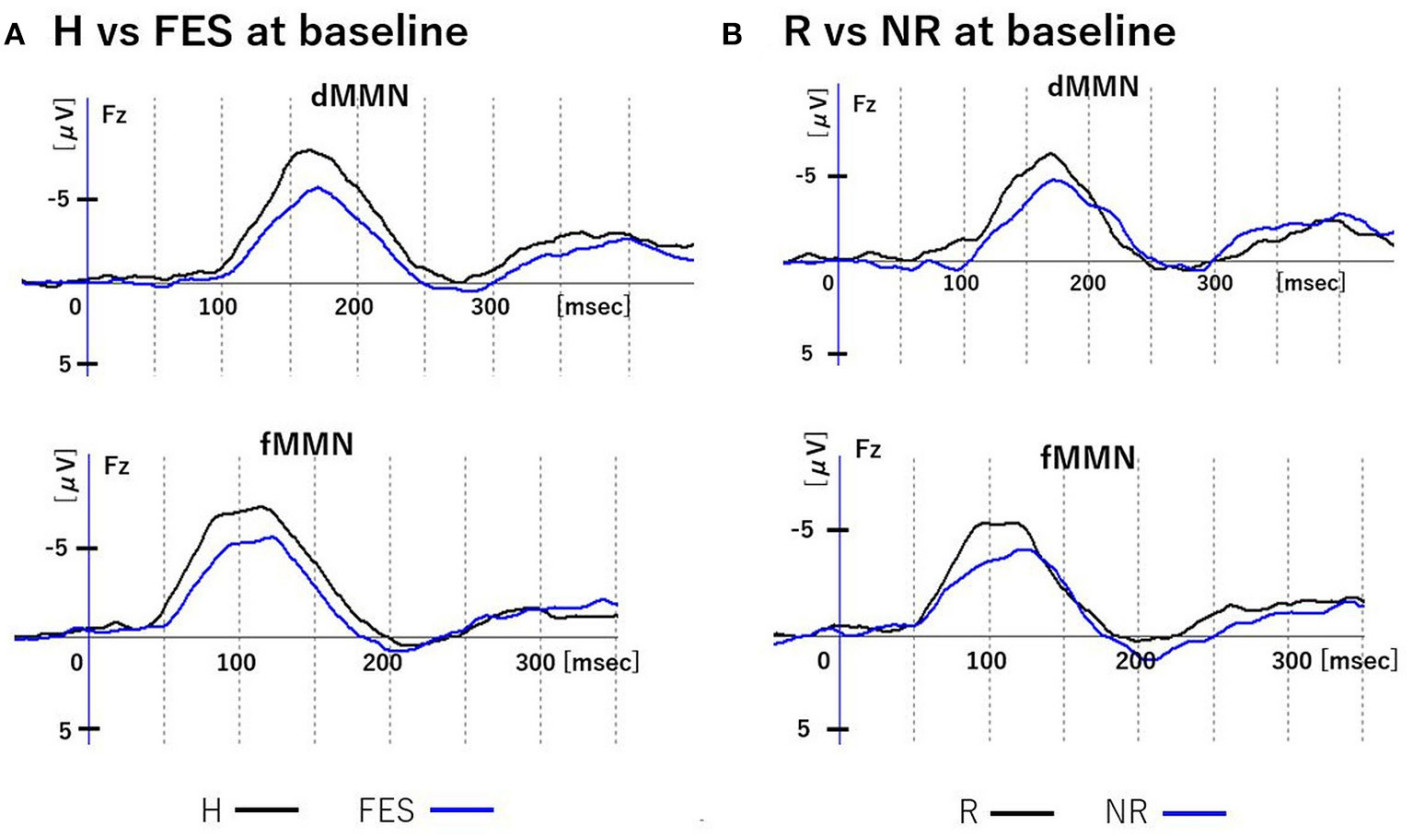
MMN waveforms at baseline. Figure shows grand average waveforms of dMMN and fMMN at Fz. **(A)** shows the waveforms of the H and FES groups. **(B)** shows the waveforms of the R and NR groups. Abbreviations: dMMN, duration mismatch negativity; fMMN, frequency mismatch negativity; H, healthy controls; FES, first episode schizophrenia; R, schizophrenia remitter; NR, schizophrenia non-remitter.

**Figure 3:**
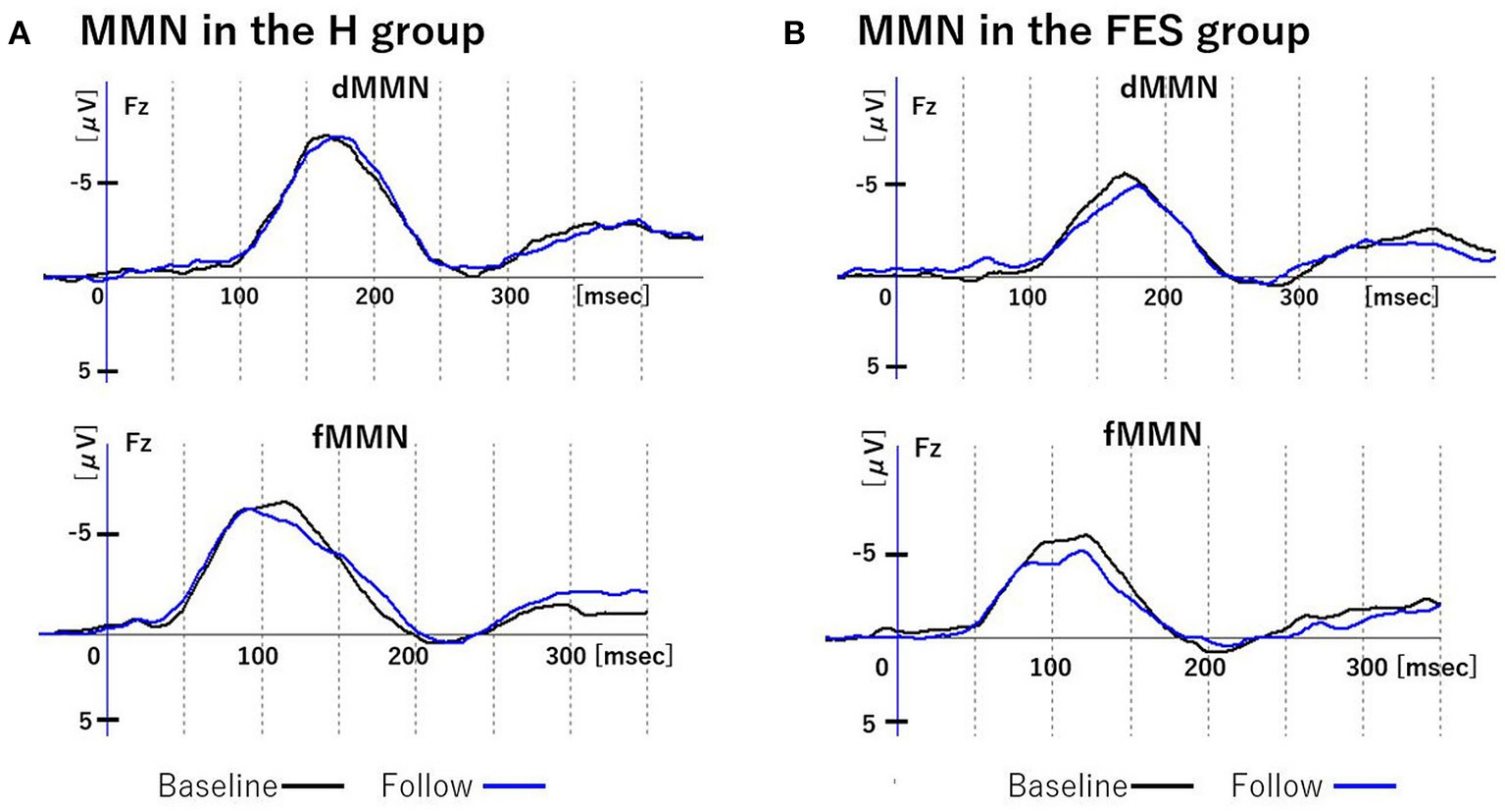
Changes in MMN over time. Figure shows grand average waveforms of dMMN and fMMN at Fz. **(A)** shows the baseline and follow-up of MMN in group H. **(B)** shows the baseline and follow-up of MMN in group FES. Abbreviations: dMMN, duration mismatch negativity; fMMN, frequency mismatch negativity; H, healthy controls; FES, first episode schizophrenia; R, schizophrenia remitter; NR, schizophrenia non-remitter.

**Figure 4:**
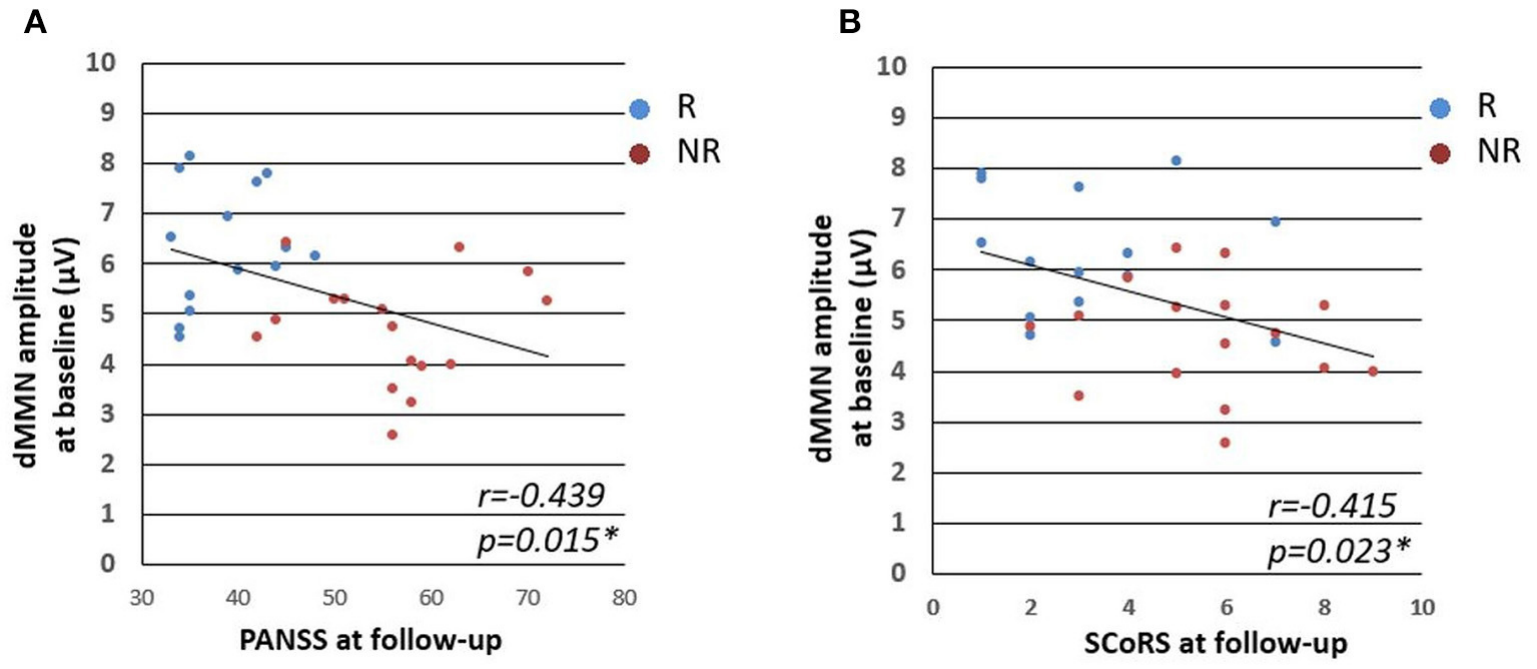
Relationships between dMMN amplitude at baseline and clinical, cognitive, and social parameters. Figures represent PANSS total score at follow up vs. dMMN amplitude at baseline **(A)** and SCoRS at follow-up vs. dMMN amplitude at baseline **(B)**. Abbreviations: R, schizophrenia remitter; NR, schizophrenia non-remitter; dMMN, duration mismatch negativity; PANSS, Positive and Negative Syndrome Scale; SCoRS, Schizophrenia Cognition Rating Scale.

Statistical Package for Social Sciences (SPSS) version 25 (SPSS Japan Inc.) and Statistica version 10 (Statsoft Inc.) were used for statistical analyses. Demographic and clinical data (Tables 1, 2) were compared between groups using a Chi-square test, or two-tailed Student's *t*-test. As shown in Figures 2, 3, the polarities of the MMN amplitudes were minus in all subjects, but their absolute values were used in the statistical analysis and in depicting Table 3 and Figures 1, 4. MMN parameters and cognitive and social function measurements at Time 1 were subtracted from those at Time 2 and defined as “Change”. Analysis of covariance (ANCOVA) was used to assess group differences in MMN parameters at Time1 and “change” between Time1 and Time2, with group (H vs. FES, R vs. NR) as a between-subject variable and age as a covariate. We used parametric statistics due to normal distribution (tested by Shapiro–Wilk test) and homogeneity (tested by Levene's test) of the variances. However, we also performed non-parametric Mann-Whitney U test for these group comparisons because of the small number of participants; the results of the study did not change except for baseline comparison of fMMN amplitude between H and FES. Time-by-group interaction in longitudinal MMN changes was also tested using repeated measures ANCOVA with group (H vs. FES, R vs. NR) and time (Time 1, Time 2) as between-subject variables and age and follow-up period as covariates.

**Table 3:** MMN parameters.

		**dMMN**				**fMMN**			
**H**	**FES**	**R**	**NR**	**H**	**FES**	**R**	**NR**
Baseline (Time 1)	amplitude [μV]	7.25 (1.52)	5.46 (1.40)	6.35 (1.16)	4.68 (1.05)	7.04 (2.20)	5.94 (1.48)	6.56 (1.15)	5.39 (1.47)
	latency [msec]	169.00 (19.59)	177.07 (20.86)	166.57 (6.35)	186.25 (19.91)	103.45 (18.60)	106.87 (21.01)	105.00 (15.80)	108.50 (23.99)
Follow (Time 2)	amplitude [μV]	7.18 (1.50)	4.81 (1.44)	5.51 (1.57)	4.21 (0.89)	6.51 (1.48)	5.05 (1.60)	5.57 (1.61)	4.62 (1.41)
	latency [msec]	169.55 (15.74)	176.40 (19.49)	178.57 (20.57)	174.50 (17.61)	104.64 (21.24)	103.72 (18.30)	108.15 (15.50)	100.12 (19.01)
Change	Δamplitude [μV]	−0.06 (1.31)	−0.65 (0.94)	−0.84 (0.83)	−0.48 (0.97)	−0.53 (2.07)	−0.91 (1.37)	−1.08 (1.44)	−0.77 (1.24)
	Δlatency [msec]	0.55 (20.43)	−0.67 (20.28)	12.00 (14.44)	−11.75 (17.32)	1.18 (18.72)	−3.38 (21.87)	2.77 (15.22)	−8.37 (24.35)

*dMMN, duration mismatch negativity; fMMN, frequency mismatch negativity; H, healthy controls; FES, first episode schizophrenia; R, schizophrenia remitter; NR, schizophrenia non-remitter. MMN data represent peak amplitudes [μV] and latencies [msec] for each group [mean (SD)]*.

Relationships between the MMN parameters at Fz and clinical variables (PANSS, mGAF, SCoRS, and BACS scores) were examined using Pearson's correlation coefficients in the combined (R + NR) patient group at both Time 1 and Time 2. We also examined whether baseline MMN parameters were associated with clinical variables at Time 2 as well as score changes of these clinical variables (Time 2–Time 1); Pearson's partial correlation coefficients with baseline clinical scores as controlling factors were used for the latter analyses.

Binary logistic regression analysis using the stepwise selection method (forward selection, likelihood ratio) was performed to investigate whether baseline characteristics including MMN parameters, neurocognitive and social functions, and clinical variables could predict remission of schizophrenia. The dependent variable was remission (R or NR). As the JART and BACS composite scores were strongly correlated (*r* = 0.438, *P* = 0.016), BACS was used as the representative covariate. As SCoRS is closely associated with mGAF (41), the latter was employed as a representative covariate. Based on these parameters, nine items were selected as covariates: MMN amplitudes (dMMN and fMMN), age, antipsychotic dosage, duration of untreated psychosis (DUP), duration of illness, PANSS total score, BACS composite score, and mGAF (at baseline).

Since there were no extreme outliers, all data were used for the statistical analyses. For all statistical analyses, the significance level was defined as *P* < 0.05.

## Results

### Characteristics of Study Population at Baseline

There were no significant differences in age, sex ratio, or follow-up period between the H and FES groups, while JART IQ was significantly lower in the FES group than in the H group (Table 1). The R and NR groups were well matched in terms of demographic and clinical variables at baseline assessment (Table 2).

### Changes in Clinical, Cognitive, and Social Function Parameters During Follow-Up

As shown in Table 2, after approximately 3 years of follow-up (Time 2), the NR group was receiving a higher dosage of antipsychotic medication compared with the R group. At Time 2, total PANSS score, each PANSS subdomain score, and RSWGcr score were higher in the NR group than those in the R group. The longitudinal changes in scores of total and each PANSS subdomain (i.e., symptom improvement) were significantly larger in the R group than those in the NR group, with the exception of the negative syndrome scale. Both R and NR groups exhibited slightly higher BACS at Time 2 relative to baseline, but no significant subgroup-by-time interaction was noted. Scores of mGAF and SCoRS at Time 2 were significantly higher in the R group than in the NR group.

### MMNs at Baseline (Time 1)

At baseline (Figures 1, 2 and Table 3), the dMMN amplitude was lower in the FES group compared to the H group [*F*_(1, 49)_ = 18.43, *P* < 0.01]. The dMMN amplitude was decreased [*F*_(1, 27)_ = 20.60, *P* < 0.01] and the dMMN latency was prolonged [*F*_(1, 27)_ = 8.10, *P* < 0.01] in the NR group compared to the R group. Non-parametric comparisons validated these group differences in the dMMN (all *P* < 0.01). In the fMMN, the amplitude was lower in the FES group compared to the H group [*F*_(1, 49)_ = 4.40, *P* = 0.04], while this difference was not significant in the non-parametric comparison (*P* = 0.07). The R and NR groups did not differ in the fMMN amplitude. The fMMN latency also showed no difference between groups.

### Longitudinal MMN “Changes” (From Time 1 to Time 2)

Parametric and non-parametric comparisons showed no significant group differences (H vs. FES, R vs. NR) in longitudinal changes of Fz amplitude/latency for both dMMN and fMMN (Figures 1, 3 and Table 3). Supplementary analyses using repeated measures ANCOVA showed no significant time-by-group interactions or main effects of time for both dMMN and fMMN. Thus, neither the dMMN nor fMMN parameters changed over time from Time 1 to Time 2 for all groups.

### Relationship Between MMN Parameters and Clinical, Cognitive, and Social Function Parameters at Time 1 and Time 2 in FES

In Time 1, there was a significant correlation between lower dMMN amplitude and lower BACS score (*r* = 0.43, *P* < 0.01), but no other correlations were found. In Time 2, lower dMMN amplitude was significantly correlated with higher PANSS (*r* = −0.43 *P* = 0.017), lower BACS (*r* = 0.53 *P* < 0.01), and lower mGAF scores (*r* = 0.42 *P* = 0.02). The fMMN did not correlate with clinical, cognitive, or social function parameters at both Time 1 and Time 2.

### Relationship Between MMN Parameters at Time 1 and Clinical, Cognitive, and Social Function Parameters at Time 2 and Their Longitudinal “Changes” in FES

Significant negative correlations were observed between dMMN amplitude at Time 1 and PANSS total score at Time 2 (r = −0.44, *P* = 0.015), and between dMMN amplitude at Time 1 and PANSS total score change (Time 2–Time 1) (*r* = −0.43, *P* = 0.024). For mGAF, SCoRS, and BACS scores (Table 4), significant relationship was identified between dMMN amplitude at Time 1 and SCoRS score at Time 2, but no significant correlation was found between BACS or mGAF and dMMN amplitude. There were no significant correlations between dMMN latency, fMMN amplitude, or fMMN latency at Time 1 and changes in these clinical, cognitive, and social function variables during follow-up (Time 2–Time 1) or those at Time 2. Collectively, the data demonstrated that a larger dMMN amplitude at Time 1 was associated with greater subsequent improvement in PANSS during follow-up period and better PANSS and SCoRS scores at Time 2 (Table 4 and Figure 4). These results did not change when DUP, antipsychotic dose at Time 1, and duration of illness were added as controlling variables.

**Table 4:** Relationships between baseline MMN parameters and clinical, cognitive, and social parameters.

		**dMMN at baseline amplitude**	
		***r***	***P***
Change	ΔPANSS: total	−0.426	0.024^*^
	ΔmGAF	0.331	0.106
	ΔSCoRS	−0.053	0.783
	ΔBACS-Composite score	−0.202	0.285
Follow-up (Time 2)	PANSS: total	−0.439	0.015^*^
	mGAF	0.251	0.180
	SCoRS	−0.415	0.023^*^
	BACS-Composite score	0.087	0.649

* *P < 0.05, Pearson correlation coefficient*.

### Logistic Regression Analysis

Binary logistic regression analysis revealed that the dMMN amplitude was positively associated with remission (OR = 0.22, 95% CI [0.065–0.747], *P* = 0.015). The result of Chi-squared test for model fit was *P* < 0.05. The Hosmer-Lemeshow test result was *P* = 0.17 and the discrimination accuracy rate was 70.8%, indicating a good fit. None of the other variables (fMMN amplitude, age, antipsychotic dosage, DUP, illness duration, PANSS score, BACS, and mGAF score) were significantly associated with remission.

## Discussion

To our knowledge, this is the first study that demonstrated in FES that lower amplitudes of baseline dMMN were significantly associated with non-remission based on RSWGcr and poor cognitive and social functions at a follow-up period of approximately 3 years later. Other factors, such as baseline demographic and clinical data and cognitive and social functions, were not identified as significant predictors of remission in patients with FES. Longitudinally, MMN did not change during follow-up period regardless of diagnosis and remission status. Thus, the present findings support the potential role of baseline dMMN as a stable biomarker that could predict symptomatic remission and improvement of cognitive and social functions in FES.

Our results highlight heterogeneity in outcomes at several years follow-up according to baseline changes in neuronal activity as encapsulated in reduced dMMN amplitude in patients with FES, where baseline dMMN amplitude may facilitate identification of individuals who are likely or less likely to achieve adequate recovery. These results expanded a previous finding in chronic schizophrenia with a short clinical follow-up (6 months) (11) and further supported a clinical utility of dMMN at earlier illness stages as a predictive marker of treatment response and recovery. Given that CHR individuals likely have reduced dMMN amplitudes, which may underpin their psychosis risk (12, 13) and functional and symptomatic improvement at follow-up period (22, 27), dMMN abnormalities may be a rather stable biomarker during the course of psychosis that could not be explained only by the effect of antipsychotic medication and/or illness chronicity after the onset. It is considered that MMN is generated by a fronto-temporal network associated with pre-attentive sensory processing (16) and that MMN reduction in schizophrenia may reflect N-methyl-D-aspartate (NMDA) receptor hypofunction (17, 18). Genetic predisposition has been hypothesized to contribute to alterations in synaptic plasticity and cortical development, predominantly by affecting NMDA receptor-mediated glutamatergic transmission, which in turn disrupts the neural circuits associated with cognitive functioning in schizophrenia (46). Thus, this mechanism may have prevented the FES patients with low baseline dMMN from achieving adequate recovery. Because other state-related characteristics associated with sensory processing (especially sensory integration) in schizophrenia, such as neurological soft signs (47) and cognitive basic symptoms (48), also contribute to poor functioning and treatment resistance (48, 49), it may be worth conducting future research to examine putative common neural underpinnings of these sensory deficits as a biomarker associated with clinical course of schizophrenia.

The fMMN amplitude at baseline was mildly decreased in the FES group compared to the H group, but there was no difference between the R and NR groups. This result may be partly explained by the notion that fMMN has less stability and replicability than dMMN (50), but it is also possible that fMMN abnormalities in schizophrenia emerge more robustly only during the chronic stages (12). Indeed, several fMMN studies in schizophrenia have demonstrated that its amplitude is associated with illness duration (51) and longitudinally declines during early illness stages (30, 52). Interestingly, such a progressive reduction on fMMN amplitude in schizophrenia was reported to have a tight coupling with ongoing gray matter atrophy in its primal generating region (i.e., Heschl's gyrus) (30). The exact mechanisms of the active brain changes after the onset remain unclear, but abnormal brain maturation (e.g., excessive synaptic pruning) (53) and glutamatergic excess due to the NMDA receptor hypofunction (54, 55) may be relevant. While the present study found no change in fMMN over time, this could be partly explained by sampling issues as described below (i.e., small sample size and relatively long illness duration of FES cohort). While dMMN may be a more static biomarker of schizophrenia than fMMN, our earlier study in CHR cohort suggested that dMMN amplitude may also exhibit longitudinal decline during transition period into psychosis (29). The study by Lho et al. also showed a decrease in dMMN of FES over time (23). Thus, future longitudinal studies in a larger cohort at various stages of psychosis would be required to clarify the specific role of MMN in the disease pathophysiology ideally using a multimodal approach (e.g., neuroimaging and biochemical investigations).

In the regression analysis, we demonstrated that dMMN amplitude was a predictor of symptomatic remission in FES, partly supporting a recent finding (28) that low amplitude of baseline dMMN was associated with treatment resistance in first-episode psychosis. However, other variables such as neurophysiological (MMN) parameters except for dMMN amplitude, various clinical variables (age, medication, illness duration, DUP, and PANSS score), and cognitive and social functions (BACS, mGAF, SCoRS, and JART IQ scores) did not contribute to the prediction. This was an unexpected finding because previous studies have demonstrated that predictors of treatment response and long-term outcome in schizophrenia included these factors (6, 11), especially DUP (4, 5). However, the present results may be partly consistent with our previous reports that changes in ERPs including MMN were observed prior to changes in neuropsychological test results (56, 57). In this regard, the behavioral and neurocognitive functions may not be severely impaired at the early stages of schizophrenia, but dMMN may accurately predict patient condition because it reflects a latent predisposition of schizophrenia even at the premorbid stage (20, 58, 59). It should be also noted that Kim et al. (11) revealed that baseline symptom severity predicted remission in chronically medicated patients with schizophrenia, suggesting that prolonged symptomatology associated with treatment resistance would affect clinical course thereafter. In contrast, we examined the FES cohort who would easily exhibit fluctuations in symptoms with medication. In addition, several FES patients in this study had received psychopharmacological interventions before psychosis onset, according to international clinical guidelines for early psychosis (60), which might have biased the results of DUP in this study.

This study has several limitations that need to be addressed. First, the sample size was relatively small, which limited the statistical power and restricted the generalizability of our results. Second, there was a significant group difference in premorbid IQ (H > NR and R), which could influence MMN in both healthy individuals and patients with psychotic disorders (61). Furthermore, the number of available EEG epochs for MMN recordings was smaller in the patients potentially due to movement artifacts (H > NR and R). However, there was no difference in IQ and MMN epoch number between the R and NR groups; hence, the essential findings of this study were unlikely to have been affected. Third, at baseline, most patients with FES were taking antipsychotics and/or other psychotropic drugs such as benzodiazepines. Although MMN amplitudes are unlikely to be affected by these medications (62, 63), our results should be replicated in patients with FES who are not taking medication. It should also be noted that our FES cohort had a rather long illness duration (up to 2 years). We failed to detect progressive decline of MMN amplitudes specific to FES, but the possibility exists that active MMN changes predominantly occur at earlier illness stages. Finally, the results of this study are limited to outcomes after approximately 3 years. In addition, because the present FES patients were not regularly assessed by PANSS, we could not assess whether baseline MMN predicts the time to remission. Further observational studies with more detailed clinical data are required to provide insight into longer-term remission.

In conclusion, the present MMN study using both cross-sectional and longitudinal designs supported that baseline dMMN amplitude of FES patients could be predictive of both symptomatic remission and cognitive and social functions.

## Data Availability Statement

The raw data supporting the conclusions of this article will be made available by the authors, without undue reservation.

## Ethics Statement

The studies involving human participants were reviewed and approved by the Committee on Medical Ethics of Toyama University. Written informed consent to participate in this study was provided by the participants' legal guardian/next of kin.

## Author Contributions

SNa was responsible for data analyses and manuscript preparation. YH, SNa, and TTat were involved in data analyses. SNa, YH, and TTat were involved in MMN data collection. DS, YM, and SNi were involved in the clinical and neuropsychological data collection. MS and TTak were involved in aspects of the study design. YH was involved in all aspects of the project, including data collection, study design, data analyses, and quality control, and checked the development of this manuscript. All authors contributed to the article and approved the submitted version.

## Funding

This study was supported by the Japan Society for the Promotion of Science KAKENHI (grant numbers 18 K07550, 16 K10205, 20H03598, and 26461739) and Japan Agency for Medical Research and Development (grant number JP19dk0307029). The funding sources were not involved in the study design; collection, analysis, and interpretation of data; writing of the report; or the decision to submit the article for publication.

## Conflict of Interest

The authors declare that the research was conducted in the absence of any commercial or financial relationships that could be construed as a potential conflict of interest.

## Publisher's Note

All claims expressed in this article are solely those of the authors and do not necessarily represent those of their affiliated organizations, or those of the publisher, the editors and the reviewers. Any product that may be evaluated in this article, or claim that may be made by its manufacturer, is not guaranteed or endorsed by the publisher.
